# Lipopolysaccharide O1 Antigen Contributes to the Virulence in *Klebsiella pneumoniae* Causing Pyogenic Liver Abscess

**DOI:** 10.1371/journal.pone.0033155

**Published:** 2012-03-12

**Authors:** Pei-Fang Hsieh, Tzu-Lung Lin, Feng-Ling Yang, Meng-Chuan Wu, Yi-Jiun Pan, Shih-Hsiung Wu, Jin-Town Wang

**Affiliations:** 1 Department of Microbiology, National Taiwan University College of Medicine, Taipei, Taiwan; 2 The Institute of Biological Chemistry, Academia Sinica, Taipei, Taiwan; 3 Department of Internal Medicine, National Taiwan University Hospital, Taipei, Taiwan; Université d'Auvergne Clermont 1, France

## Abstract

*Klebsiella pneumoniae* is the common cause of a global emerging infectious disease, community-acquired pyogenic liver abscess (PLA). Capsular polysaccharide (CPS) and lipopolysaccharide (LPS) are critical for this microorganism's ability to spread through the blood and to cause sepsis. While CPS type K1 is an important virulence factor in *K. pneumoniae* causing PLA, the role of LPS in PLA is not clear. Here, we characterize the role of LPS O antigen in the pathogenesis of *K. pneumoniae* causing PLA. NTUH-K2044 is a LPS O1 clinical strain; the presence of the O antigen was shown via the presence of 1,3-galactan in the LPS, and of sequences that align with the *wb* gene cluster, known to produce O-antigen. Serologic analysis of *K. pneumoniae* clinical isolates demonstrated that the O1 serotype was more prevalent in PLA strains than that in non-tissue-invasive strains (38/42 vs. 9/32, *P*<0.0001). O1 serotype isolates had a higher frequency of serum resistance, and mutation of the O1 antigen changed serum resistance in *K. pneumoniae*. A PLA-causing strain of CPS capsular type K2 and LPS serotype O1 (i.e., O1:K2 PLA strain) deleted for the O1 synthesizing genes was profoundly attenuated in virulence, as demonstrated in separate mouse models of septicemia and liver abscess. Immunization of mice with the K2044 *magA*-mutant (K_1_^−^ O_1_) against LPS O1 provided protection against infection with an O1:K2 PLA strain, but not against infection with an O1:K1 PLA strain. Our findings indicate that the O1 antigen of PLA-associated *K. pneumoniae* contributes to virulence by conveying resistance to serum killing, promoting bacterial dissemination to and colonization of internal organs after the onset of bacteremia, and could be a useful vaccine candidate against infection by an O1:K2 PLA strain.

## Introduction

Community-acquired pyogenic liver abscess (PLA) is an emerging infectious disease. According to recent epidemiologic studies, 80% of cases of PLA were caused by *Klebsiella pneumoniae*; 60% to 80% of the *K. pneumoniae* isolates causing these cases belonged to the K1 capsular type, and 10% to 14% isolates belonged to the K2 capsular type in Asia [1], [2]. Most PLA-associated *K. pneumoniae* strains, but not strains that are not tissue-invasive, show a hypermucoid phenotype, serum resistance, and resistance to phagocytosis [3]. Capsular polysaccharide (CPS) has been shown to be essential for the virulence of this pathogen [3]. *magA* is predicted to be a capsular polymerase gene *wzy* for K1 by sequence alignment. Mutations in *mag*A have been shown to result in CPS deficiency and avirulence in a mouse model of septicemia [3], [4].

As the outermost components of the bacterial surface, CPS and the O-antigen portion of the lipopolysaccharide (LPS) are among the first bacteria-derived molecules to be encountered by the host's innate immune system. Both CPS and LPS components are important pathogenic determinants in *K. pneumonia*-caused pneumonia and bacteremia [5]–[7], but little is known about the virulence role of LPS in *K. pneumoniae* PLA.

LPS consists of three structural domains: lipid A, the core oligosaccharide (OS), and the O antigenic polysaccharide (O-PS). The O antigen is the outermost component of LPS and consists of a polymer of oligosaccharide repeating units. Among clinical *K. pneumoniae* isolates, the O1 antigen is the most common O antigen [8]. The clinically prevalent O1 antigen contains two structurally distinct O-PS domains composed of the repeat units d-galactan I and d-galactan II. O-antigen biosynthesis is performed by the products of the *wb* (*rfb*) gene cluster; the high chemical variability shown by O antigens is reflected by the genetic variation in the corresponding genes [9], [10]. Genes of the *wb* cluster in *K. pneumoniae* O1 are required for the expression of d-galactan I [11], [12], but the locations and identities of genes required for d-galactan II biosynthesis remains unknown. The *wb* gene cluster contains six genes. The *wzm* and *wzt* (formerly *rfbAB*) loci encode the transmembrane and ATP-binding components of the ABC-2 transporter, respectively [13]. *glf* encodes a UDP-galactopyranose mutase, which generates uridine 5′-diphospho-α-d-galactofuranose (UDP-Galf), the biosynthetic precursor of galactofuranosyl residues [14]. The remaining three genes (*wbbM*, *wbbN*, and *wbbO*) encode three galactosyltransferases and form a membrane-localized glycosyltransferase complex [15]. *wbbO* is the last gene of the *wb* cluster; the WbbO gene product is the first dedicated enzyme in the assembly pathway for the O1 antigen [16].

In the present study, we examined the distribution of O1 serotypes in PLA and non–tissue-invasive *K. pneumoniae* clinical isolates; determined whether a correlation exists between O1 serotype and resistance to killing by serum; explored the role of the O1 antigen of *K. pneumoniae* in the pathogenesis of PLA; and investigated whether antiserum raised against LPS O1 could protect against PLA-associated *K. pneumoniae* infection.

## Results

### NTUH-K2044 is a LPS O1-type strain

We isolated and analyzed the LPS of a clinical *K. pneumoniae* PLA strain, NTUH-K2044, and a corresponding *magA* (CPS-deficient) mutant. Based on LPS composition, the major sugar was galactose; no glucosamine or mannose was detected. The major sugar linkage was 1,3-galactosidic. The detailed chemical structure of O antigens of the serotype (O1) has been identified as repeated D-1,3-galactan polymers [12], [17] Separately, analysis (using Basic Local Alignment Search Tool (BLAST)) of the nucleotide sequences of the *wb* cluster from NTUH-K2044 revealed 98.3% sequence identity compared with the cluster from the 889/50 strain (O1:K20), but only 73.4% sequence identity compared with the genes from the CWK47 strain (O8) [11], [18]. Gene order (synteny) and sequence similarity are poorer in comparison to the *wb* clusters of isolates of other LPS serotypes. Both results (chemical analysis of LPS and sequence analysis of *wb*) indicate that NTUH-K2044 is a LPS O1-type strain.

### Reactivity of antiserum from magA-mutant hyperimmune mice against LPS O1

**Figure 1:**
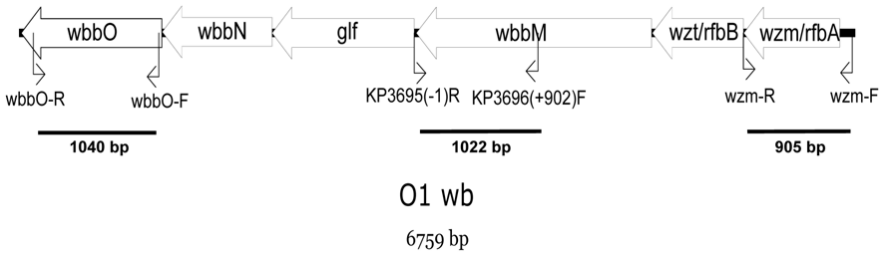
Genetic organization of the *wb* cluster in serotype O1 *K. pneumoniae* strain. The PCR primer alignments and the lengths of the amplicons (*wbbO*, *KP3695/wbbM*, and *wzm*) used to study the frequency of O1 type in the *K. pneumoniae* strains are shown. Arrows denote the orientations of the open-reading frames.

**Figure 2:**
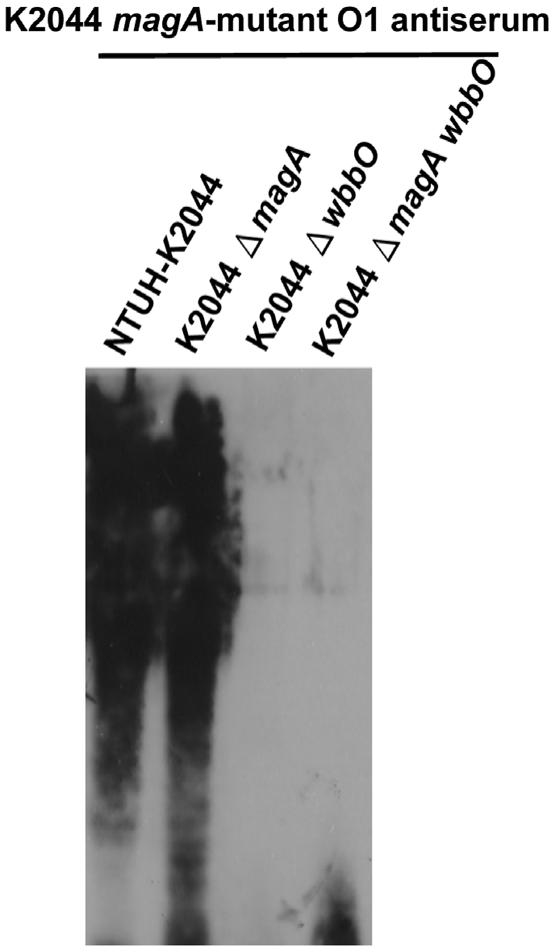
Specificity of O1-antiserum from K2044 *magA* mutant hyperimmune mice. EPS were prepared from *K. pneumoniae* NTUH-K2044, the K2044 *wbbO* mutant, the K2044 *magA* mutant, and the K2044 *magA wbbO* double mutant strain. Extracts from normalized bacterial suspensions (10^8^ CFU) were separated by 12% SDS-PAGE and visualized by immunoblot.

In order to determine the prevalence of the LPS O1-type in clinical isolated *K. pneumoniae* strains, 3 primer pairs were designed for the polymerase chain reaction (PCR) detection of O1-serotype-synthesizing genes. Because the LPS O types of the 77 K-antigen *Klebsiella* reference strains were determined previously [8], the conserved sequences of *wbbO*, *KP3695/wbbM*, and *wzm* from the 889/50 strain (O1:K20) and the NTUH-K2044 strain (O1:K1) were used to target the corresponding domains of the O1-serotype *wb* cluster of K-antigen *Klebsiella* reference strains (Figure 1). Among 77 K-antigen *Klebsiella* reference strains, we found that 63% to 73% (19/30 to 22/30) of the O1-serotype isolates and 23% (11/47) of the non–O1-serotype isolates were identified by PCR with these primer pairs (Table 1). The primers designed for detection of the LPS O1-type showed a limited sensitivity and specificity. Next, we generated antiserum which was supposed to be against LPS O1 by immunization of the unencapsulated K2044 *magA* mutant (K_1_^−^ O_1_) in mice. The reactivity to LPS O1 of the K2044 *magA*-mutant hyperimmune antiserum was evaluated by immunoblotting against the exopolysaccharide (EPS) extracts of K-antigen *Klebsiella* reference strains. Antiserum against the K2044 *magA* mutant reacted with all of 20 randomly selected O1-serotype isolates but with none of 20 randomly selected isolates known to belong to other serotypes or that were not O-antigen typeable. Therefore, the antiserum demonstrated 100% (20/20) sensitivity and 100% (20/20) specificity against O1-type strains. Immunoblotting was also performed against EPS extracts of the K2044 *wbbO* mutant (K_1_ O_1_^−^) and K2044 *magA wbbO* double mutant (K_1_^−^O_1_^−^). No cross-reaction was seen for either mutant (Figure 2), demonstrating that antiserum generated from the K2044 *magA*-mutant hyperimmune mice is specific to LPS O1.

**Table 1:** LPS O1-type *wb* PCR of proposed O antigen type of the 77 K-antigen *Klebsiella* reference strains.

Serotype*	Number of tested strains	O1-*wb* gene		
		wbbO (1040 bp)	wbbM (1022 bp)	wzm (905 bp)
O1	30	22	19	21
O2	10	5	5	5
O2ac	2	1	2	2
O3	13	0	0	0
O4	3	0	0	0
O5	5	0	0	0
O7	1	0	0	0
O8	1	0	0	0
O12	1	0	0	0
NT O^−^	6	3	3	3
NT O^+^	5	2	1	1
Sensitivity (%)		73.3	63.3	70.0
Specificity (%)		75.3	71.4	74.0

* NT, nontypeable.

### Prevalence of O1 antigen in *K. pneumoniae* clinical isolates

We compared the distribution of O1 serotypes by immunoblotting of samples from 74 clinical isolates of *K. pneumoniae*, including 42 PLA strains and 32 non-tissue-invasive strains. The O1 serotype accounted for 63.5% of all clinical isolates, with significant differences in representation among the strains that caused PLA (38/42; 90.5%) and those that were not tissue-invasive (9/32; 28.1%) (*P*<0.0001, chi-square test).

### Resistance to serum killing in O1- and non–O1-serotype *Klebsiella* strains

**Figure 3:**
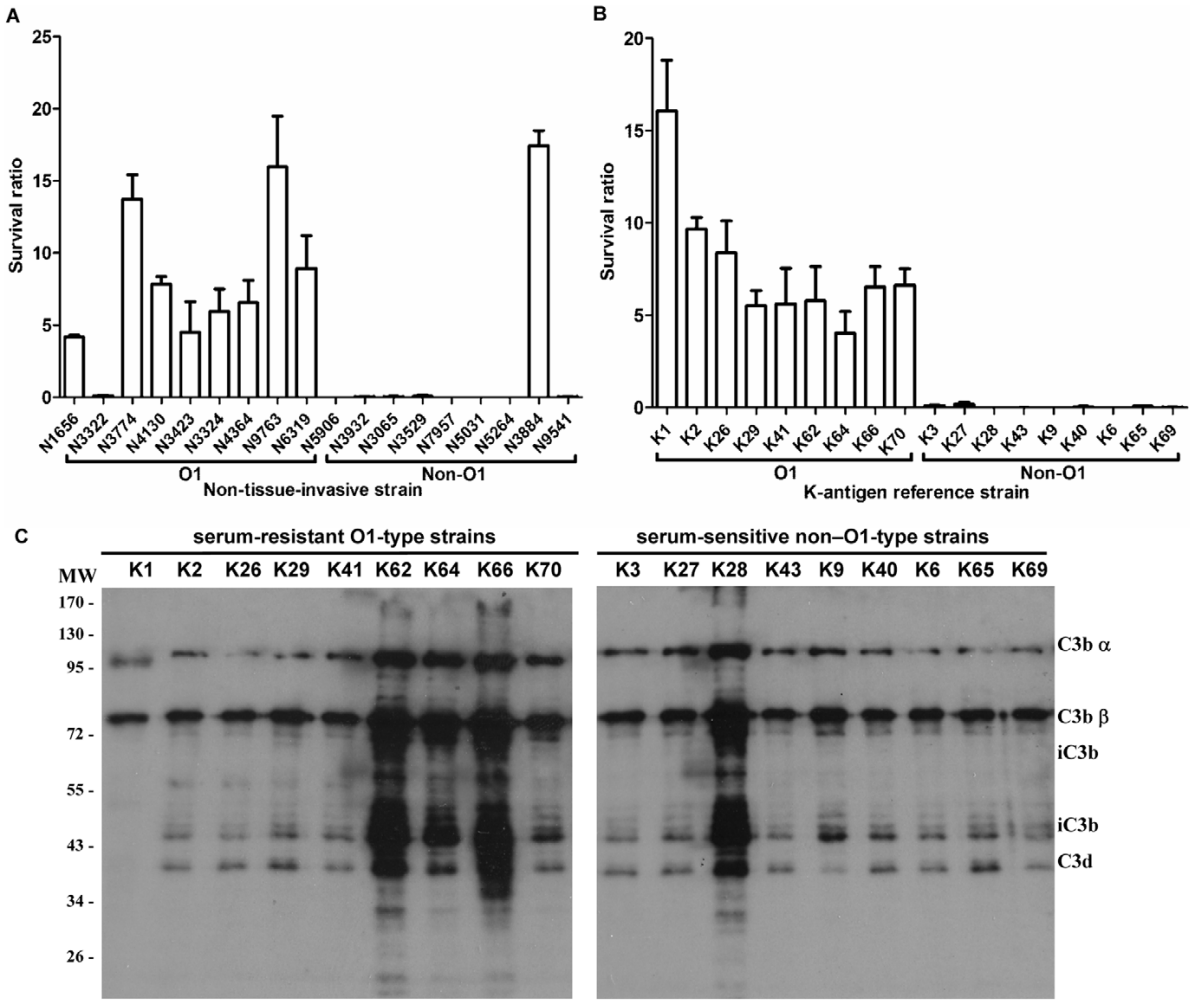
Sensitivities of the O1-type and non–O1-type *Klebsiella* strains to serum killing and complement C3 deposition. ***A*** **and** ***B***, serum sensitivity assays. Differences in resistance to killing by nonimmune healthy human serum between the *K. pneumoniae* clinical isolate non-tissue-invasive and *Klebsiella* K-antigen reference strains. The data represent the means of three independent trials; the error bars represent the standard deviations. An average survival ratio ≥1 corresponds to serum resistance. ***C***, Complement C3 deposition on *Klebsiella* K-antigen reference strains. Western blot analysis of complement C3 deposition on 9 serum-resistant O1-type and 9 serum-sensitive non–O1-type *Klebsiella* strains. Samples were exposed to serum for 1 min.

Previous study reported that the prevalence of the O1 serotype was higher among serum-resistant strains than among serum-sensitive strains of *K. pneumoniae* clinical isolates [19]. Among our *K. pneumoniae* clinical isolates, 8 of 9 non–tissue-invasive O1 serotype strains were resistant to killing by serum, whereas 1 of 9 non–tissue-invasive strains which was not O1 serotype was resistant to serum. Among 18 randomly selected *Klebsiella* K-antigen reference strains, 9 of 9 O1-serotype strains were resistant to serum. In contrast, 0 of 9 strains which was not O1 serotype was resistant. The frequency of serum-resistant strains among the isolates which were of serotype O1 (94.4%; 17/18 strains) was significantly higher than that among the isolates which were not of serotype O1 (5.6%; 1/18 strains) (*P*<0.0001, chi-square test, Figure 3A and 3B). Therefore, our data showed consistent with the previous finding and a higher frequency of serum resistance among the O1-serotype *Klebsiella* isolates than among non-O1-serotype *Klebsiella* isolates. We also examined the deposition of complement C3 in these tested reference strains. However, no direct correlation between the C3 deposition pattern and bacterial resistance was observed in serum-resistant O1-type strains and serum-sensitive non–O1-type strains (Figure 3C).

### Characterization of the O1 deletion mutant

**Figure 4:**
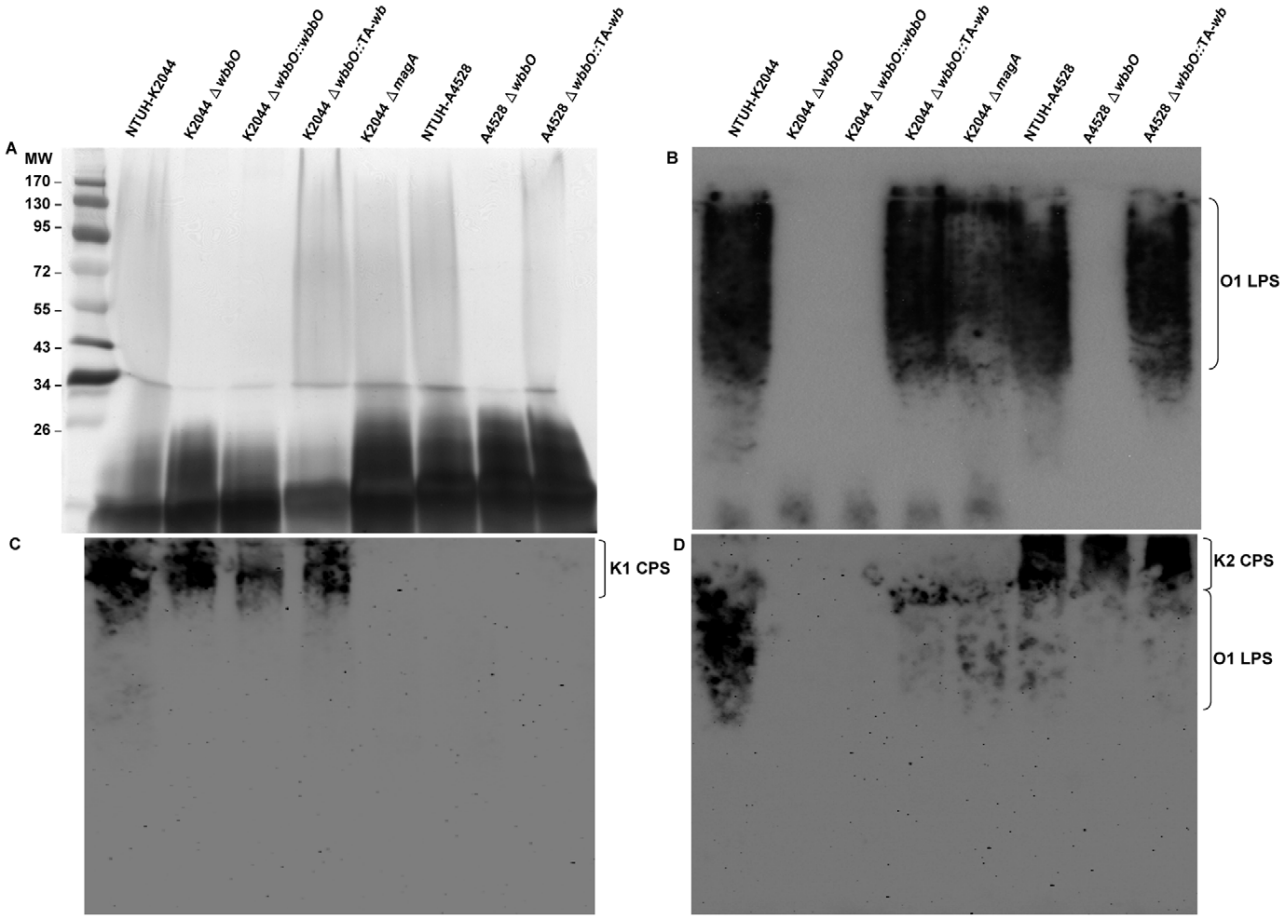
EPS phenotype of *K. pneumoniae* NTUH-K2044 (O1:K1), NTUH-A4528 (O1:K2), and the corresponding *wbbO* mutant and *wbbO* complementation strains. EPS were prepared from the *K. pneumoniae* NTUH-K2044, NTUH-A4528, and *wbbO* mutants and *wbbO* complementation strains in the respective genetic backgrounds. Extracts from normalized bacterial suspensions (10^8^ CFU) were separated by 12% SDS-PAGE and visualized by silver staining or immunoblotting. ***A*** and ***B***, Silver staining and immunoblotting with K2044 *magA* mutant hyperimmune O1 antiserum. ***C*** and ***D***, Immunoblots of the same material developed with rabbit anti-K1 antiserum (Statens Serum Institute; 1∶10,000) (*C*); rabbit anti-K2 antiserum (Denka Seiken, 1∶5,000) (*D*).

To generate the O1-antigen deficient mutant, a *wbbO* mutant of NTUH-K2044 (O1:K1) was constructed (knock-out) by unmarked deletion. We previously reported [20] that the chemical structure of the CPS of *K. pneumoniae* NTUH-K2044 consisted of a repeated trisaccharide [→3]-β-d-Glc-(1→4)-[2,3-(*S*)-pyruvate]-β-d-GlcA-(1→4)-α-l-Fuc-(1→). Chemical structure analysis of the EPS of the K2044 *wbbO*-mutant strain revealed the presence of CPS sugars, such as fucose, glucose and glucuronic acid; galactose and 1,3-galactosidic linkages were not seen. Moreover, compared to the parent strain, the K2044 *wbbO* mutant had lost its O antigen, as determined by silver staining and by immunoblotting (Figure 4A and 4B). Introduction into the *wbbO* mutant of a copy of the *wbbO* gene under control of its own promoter did not restore the O antigen production. However, the O antigen production was restored partially by complementation with the plasmid containing the entire *wb* cluster (Figure 4A and 4B). Since the *wb* cluster contains the promoter for the *wb* operon and six genes, *wzm*, *wzt*, g*lf*, *wbbM*, *wbbN*, and *wbbO*, this result proper regulation of the *wbbO* gene expression requires cis-elements within the *wb* cluster. We obtained similar results with another clinically isolated O1-type A4528 *wbbO*-mutant (K_2_ O_1_^−^) strain (Figure 4A and 4B). Anti-K1 reactivity was against EPS extracts of NTUH-K2044 (O1:K1) derivatives, but not against that of the unencapsulated K2044 *magA* mutant or that of NTUH-A4528 (O1:K2) derivatives. Surprisingly, anti-K2 reactivity was observed not only against the EPS extracts of NTUH-A4528 (O1:K2) derivatives, but also against the EPS extracts of NTUH-K2044 (O1:K1) derivatives (Figure 4C and 4D). Apparently, the anti-K2 sera (raised against formalin-killed whole O1:K2 bacteria) recognized both the K2 antigen and the O1 antigen (Figure 4D).

**Figure 5:**
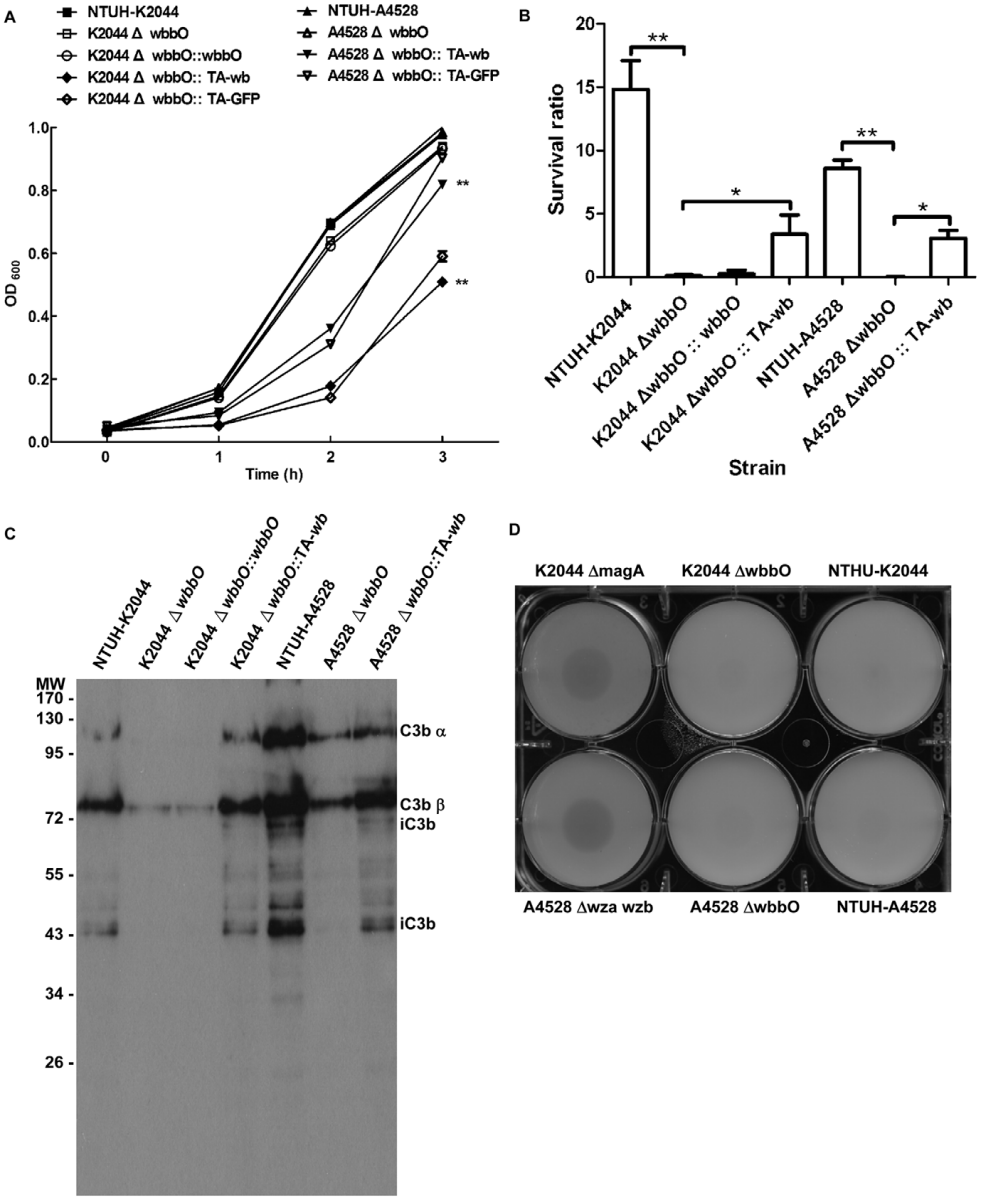
Sensitivities of the *wbbO*-mutant strains to serum killing, complement C3 deposition, and phagocytosis by *Dictyostelium*. ***A***, Growth rates in LB broth of the *K. pneumoniae* wild-type, *wbbO* mutants, *wbbO* complementation strains, and the *wbbO* mutants with the GFP expression plasmid strains . The data represent the means of three independent trials; the error bars represent the standard deviations. ^**^ *P*<0.01 by Student's *t* test (comparing the *wb* cluster-complemented *wbbO* mutant vs. the wild-type or *wbbO* mutant strains). ***B***, Serum sensitivity assays of resistance to killing by nonimmune healthy human serum, comparing the wild-type, the *wbbO* mutant, and *wbbO* complementation strains. The data represent the means of three independent trials; the error bars represent the standard deviations. The average survival ratio ≥1 corresponds to serum resistance. ^**^ *P*<0.01 or ^*^ *P*<0.05 by Student's *t* test (comparing *wbbO* mutant strains vs. the wild-type or the *wb* cluster-complemented *wbbO* mutant). ***C***, Western blot analysis of complement C3 deposition. Samples were exposed to serum for 1 min. ***D***, Resistance to phagocytosis in the *K. pneumoniae* wild-type and *wbbO* mutant strains by plaque assays. Pictured assay used 5000 *Dictyostelium* cells per test. Non-encapsulated mutants were permissive for *Dictyostelium* growth and were used as positive controls.

To further characterize the phenotypic changes in the *wbbO* mutant in comparison with the wild-type strain, some *in vitro* assays were performed. The O1 deletion mutants exhibited a mucoid phenotype and produced amounts of CPS similar to that of the NTUH-K2044 or the NTUH-A4528 wild-type strains (Table 2 and Table 3). As assayed in LB broth culture, the growth rate of the *wbbO* mutants showed no significant difference compared to that of the wild-type strains. In contrast, growth of the *wbbO* mutant was slower upon introduction of the *wb* cluster complementation plasmid, but this effect was attributable to carriage of the replicative TA-*wb* plasmid, since similar effects were seen upon introduction of a TA-GFP expression plasmid (Figure 5A).

**Table 2:** The capsule quantification of *K. pneumoniae* NTUH-K2044, NTUH-A4528, *wbbO* mutants and *wbbO* complementation strains in the respective genetic backgrounds and non-encapsulated mutants.

Strain	Quantity (mean ± S.D.)*	-Fold†
NTUH-K2044	19.62±1.47	5.60
K2044 ΔwbbO	19.75±0.09	5.63
K2044 ΔwbbO::wbbO	19.03±0.94	5.43
K2044 ΔwbbO::TA-wb	19.27±0.32	5.49
K2044 ΔmagA	3.51±0.47	1.00
NTUH-A4528	15.93±0.79	4.30
A4528 ΔwbbO	15.43±0.59	4.17
A4528 ΔwbbO::TA-wb	15.72±0.28	4.24
A4528 Δwza wzb	3.70±0.65	1.00

* Values are the averages of triplicate samples and are given as micrograms of uronic acid/10^9^ CFU.

† Comparison based on the non-capsulated strain K2044 Δ*magA* or A4528 Δ*wza wzb*.

**Table 3:** Phenotypic characterization of *K. pneumoniae* NTUH-K2044, NTUH-A4528, *wbbO* mutants and *wbbO* complementation strains in the respective genetic backgrounds and non-encapsulated mutants.

Strain	Genotype or phenotype*	CPS K Ag	LPS O1 Ag	Serum resistance†	LD_50_ values (CFU) of intraperitoneal inoculation
NTUH-K2044	wild-type/m^+^	K1^+^	+	R	<1×10^2^
K2044 ΔwbbO	*wbbO*/m^+^	K1^+^	−	S	1×10^3^
K2044 ΔwbbO::TA-wb	wild-type/m^+^	K1^+^	+	R	7.5×10^2^
K2044 ΔmagA	*magA*/m^−^	K1^−^	+	S	>1×10^7^
K2044 ΔmagA wbbO	*magA wbbO*/m^−^	K1^−^	−	S	ND
NTUH-A4528	wild-type/m^+^	K2^+^	+	R	<1×10^2^
A4528 ΔwbbO	*wbbO*/m^+^	K2^+^	−	S	2.8×10^4^
A4528 ΔwbbO::TA-wb	wild-type/m^+^	K2^+^	+	R	2.2×10^3^
A4528 Δwza wzb	*wza wzb*/m^−^	K2^−^	+	ND	>1×10^7^

* m^+^, mucoid phenotype; m^−^, non-mucoid phenotype.

† R, resistance; S, sensitive.

ND, not determined.

Both the O and K antigens were shown to contribute significantly to the virulence of *K. pneumoniae*, by conferring protection against serum killing and phagocytosis, respectively [3], [21], [22]. To test the sensitivity to the serum's bactericidal effect of the *wbbO* mutant strains, serum killing assays were performed. Killing of the *wbbO* mutants by non-immunized healthy human serum was more efficient than killing of the NTUH-K2044 or NTUH-A4528 wild-type strain, presumably because mutants lacked O1 antigen. The serum-sensitive phenotype in the *wbbO* mutants could be partially restored by complementation with the *wb* cluster (Figure 5B). The levels of C3 complement components deposited on the NTUH-A4528 (O1:K2) wild-type strain were much higher than those deposited on the NTUH-K2044 (O1:K1) wild-type strain. In both backgrounds, deposition was reduced by mutation of *wbbO*, and the effect was partially counteracted by complementation with the *wb* cluster (Figure 5C). To test the opsonophagocytic activity of the mutant strains, a *Dictyostelium* model was used [22]. *wza* and *wzb*, which are located in the region coding for the K2 CPS biosynthetic machinery, were deleted in NTUH-A4528; the *wza wzb* double mutant then was assessed for virulence. As expected, these mutations resulted in the loss of K2 CPS; the mutant exhibited an avirulent phenotype in a septicemia mouse model (Table 2 and Table 3). At doses of up to 5000 *Dictyostelium* cells, the *wbbO* mutants were as resistant to phagocytosis as the respective wild-type strains (Figure 5D). In contrast, the unencapsulated K2044 *magA* or A4528 *wza wzb* mutants were more susceptible to phagocytosis by *Dictyostelium* cells (Figure 5D). Thus, the O antigen confers protection against serum killing, but not against phagocytosis.

### Virulence of O1-deletion mutant during PLA-associated *K. pneumoniae* infection

**Figure 6:**
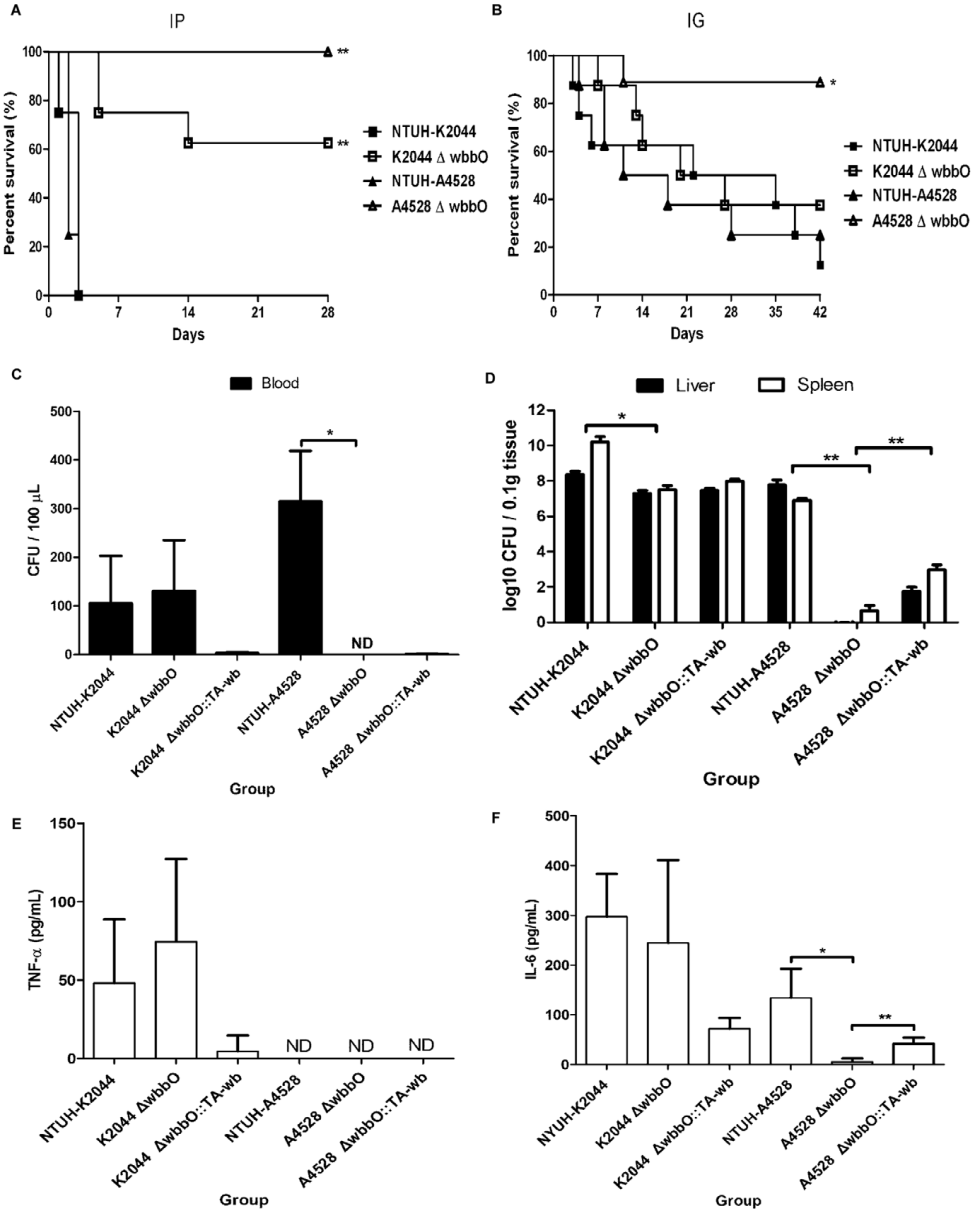
Survival rate and immune response in *K. pneumoniae*-inoculated mice. ***A*** and ***B***, Eight mice per group were infected with the wild-type and the *wbbO* mutant strains at an intraperitoneal (IP) dose of 1×10^3^ CFU/animal or an intragastrical (IG) dose of 1×10^6^ CFU/animal. Survival of mice was monitored for 4 (IP) or 6 (IG) weeks. ▪, NTUH-K2044; □, K2044 *wbbO*; ▴, NTUH-A4528; ▵, A4528 *wbbO* mutant (K2044 *wbbO* vs. parent, *P* = 0.0002 (IP) and *P* = 0.4417 (IG); A4528 *wbbO* vs. parent, *P*<0.0001 (IP) and *P* = 0.0129 (IG); log-rank test). ***C*** to ***F***, Equivalent doses (1×10^3^ CFU) of the wild-type, the *wbbO* mutant, and the *wbbO* mutant with the *wb* cluster complementation were inoculated into mice (4 mice per group) by IP injection. Surviving animals were sacrificed at 24 hours after challenge. Bacterial loads were measured in the blood (***C***), liver, and spleen (***D***); TNF-α (***E***) and IL-6 (***F***) levels were measured in the serum. Log_10_ CFU was standardized per 0.1 gram wet organ weight. IL-6 and TNF-alpha levels were measured by ELISA. Data are presented as means ± standard deviations. ND, not detected. ^**^ *P*<0.01 and ^*^ *P*<0.05 by Student's *t* test (*wbbO* mutant group vs. wild-type group or the *wbbO* mutant with the *wb* cluster complementation group).

Upon intraperitoneal (IP) infection of mice, the *wbbO* mutants had LD_50_ values of 1.0×10^3^ CFU (K2044 background) and 2.8×10^4^ CFU (A4528 background) (Table 3). Both *wbbO* mutants were less virulent than the respective parent strains (Figure 6A). Complementation of the *wbbO* mutant with the *wb* cluster partially restored virulence in mice, with LD_50_ values (via the IP route) of 7.5×10^2^ CFU and 2.2×10^3^ CFU, respectively, for the complemented strains (Table 3). When mice were challenged by intragastric infection, the survival of the mice infected with the A4528 *wbbO* mutant was increased markedly compared to that of mice infected with the A4528 parental strain. However, the survival of the mice was not significantly different for animals infected with the K2044 *wbbO* mutant compared to the respective parental strain (Figure 6B). To explore bacterial dissemination *in vivo*, we examined the bacterial load in mice challenged with equivalent doses (1×10^3^ CFU) of the wild-type strains, the *wbbO* mutant strains, and the Δ*wbbO wb* cluster complementation strains. When organs were examined in surviving animals at 24 hr after bacterial inoculation, the mice infected with the A4528 *wbbO* mutant had no detectable bacteria in the blood (*P* = 0.028, Figure 6C). In both genetic backgrounds, the *wbbO* mutants yielded significantly fewer colony counts both in the liver and spleen compared with the respective wild-type parent. In the A4528 background, complementation of the *wbbO* mutant with the *wb* cluster partially restored the colony counts in both in the liver and spleen (Figure 6D). Previous studies revealed that TRIF- and MyD88-dependent TLR signaling, which stimulate proinflammatory cytokines TNF-alpha and IL-6, contribute to host defense against *Klebsiella* infection [23]. Although the *wbbO* mutants exhibited reduced lethality in the mouse model, the mutants stimulated the proinflammatory cytokine TNF-α to levels comparable to those resulting from infection with the wild-type strains (Figure 6E). However, serum IL-6 concentrations were reduced in the mice infected with the A4528 *wbbO* mutant group compared to animals infected with the NTUH-A4528 wild-type strain (*P* = 0.011, Figure 6F). Meanwhile, infection with the *wb* cluster-complemented A4528 *wbbO* mutant stimulated IL-6 production to levels higher than those seen with the A4528 *wbbO* mutant (*P* = 0.002, Figure 6F). These data demonstrate that mutants impaired for O1 production have decreased virulence, with reduced bacterial colonization in organs and decreased host inflammatory response to *K. pneumoniae* infection.

### Protective effect of anti-O1 antibodies during *K. pneumoniae* infection

**Figure 7:**
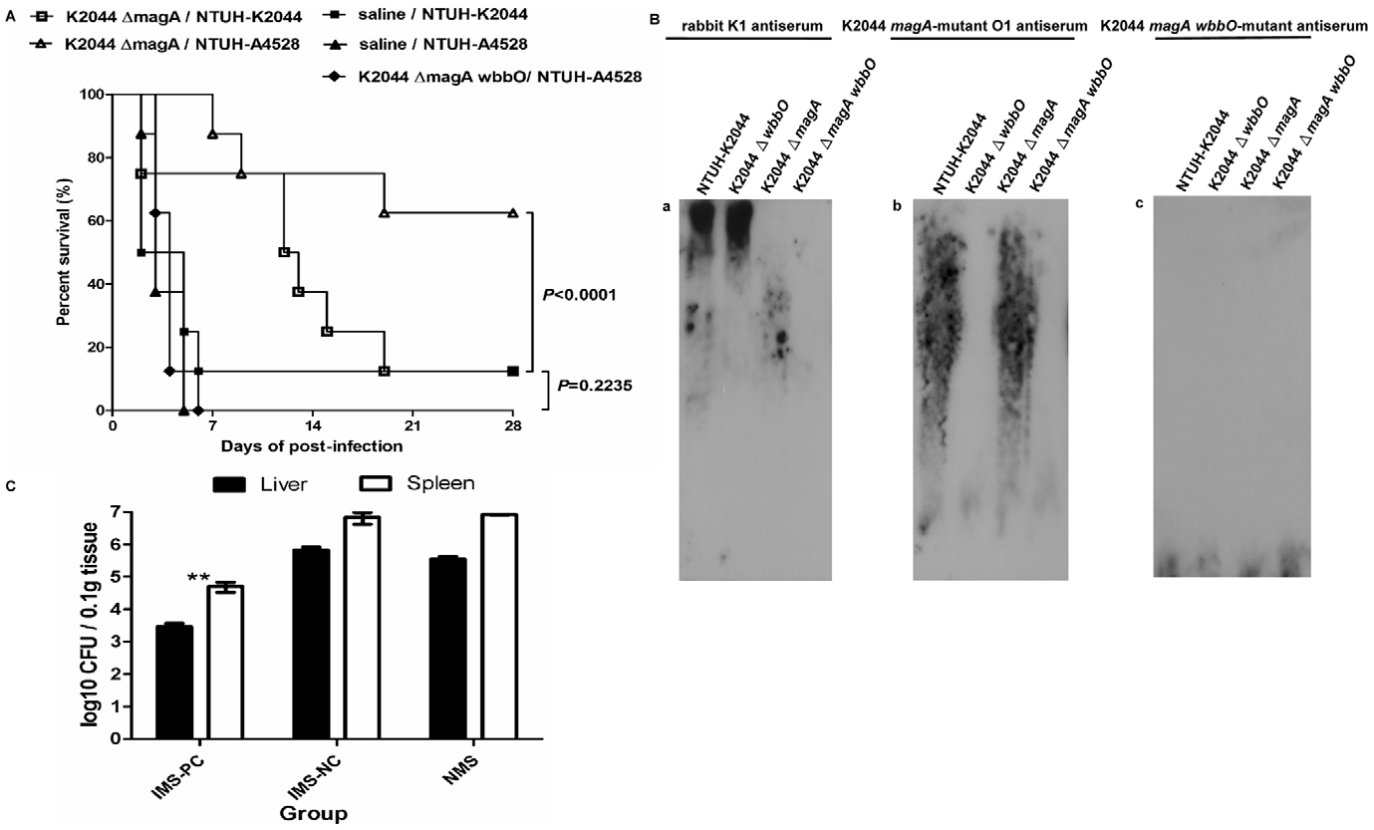
Survival rate and immune response in K2044 *magA* mutant hyperimmune and virulent K1/K2 *K. pneumoniae*-challenged mice. ***A***, Survival of K2044 *magA* mutant hyperimmune mice challenged with NTUH-K2044 (O1:K1) or NTUH-A4528 (O1:K2). Mice (8 per group) were inoculated three times by once-weekly IP injections with 1×10^6^ CFU of K2044 *magA*-mutant or K2044 *magA wbbO* double mutant. Age-matched, unimmunized control mice were inoculated with saline. At the fourth week, immunized groups were challenged with NTUH-K2044 or the NTUH-A4528 (1×10^3^ CFU per animal, IP). Survival was assessed for 28 days following infection. □, K2044 *magA* mutant hyperimmune, NTUH-K2044 challenge.; ▪, nonimmune, NTUH-K2044 challenge; ▵, K2044 *magA* mutant hyperimmune, NTUH-A4528 challenge;▴, nonimmune, NTUH-A4528 challenge; ⧫, K2044 *magA wbbO* double mutant hyperimmune, NTUH-A4528 challenge. By log-rank test: *P* = 0.2235, K2044 *magA/*NTUH-K2044 vs. nonimmune/NTUH-K2044; *P*<0.0001, K2044 *magA/*NTUH-A4528 vs. nonimmune/NTUH-A4528 or K2044 *magA wbbO*/NTUH-A4528. ***B***, Immune response of anti-O1 IgG in K2044 *magA*-mutant hyperimmune mice. Immunoblots developed with rabbit anti-K1 antiserum (purchased from Statens Serum Institute, 1∶10,000) (*a*), K2044 *magA* mutant immune mouse serum (1∶2500) (*b*) or K2044 *magAwbbO* double mutant immune mouse serum (1∶500) (*c*). ***C***, O1-Ag-specific antibodies reduce growth of NTUH-A4528 (O1:K2) *K. pneumoniae* in mice. Groups of 4 BALB/c mice were treated with one of the following: naïve mouse serum (NMS), K2044 *magA* mutant immune mouse serum (IMS–positive control, IMS-PC), K2044 *magAwbbO* double mutant immune mouse serum (IMS–negative control, IMS-NC). One hour after injection, mice were infected with 1×10^4^ CFU the NTUH-A4528 by intraperitoneal injection. Four hours after infection, the number of bacteria in the liver and spleen were determined. Log_10_ CFU was standardized per 0.1 gram wet organ weight. The black and white bars represent the means for each group for liver and spleen, respectively; error bars indicate standard deviations. ^**^ *P*<0.01 by Student's *t* test (IMS-PC vs. IMS-NC or NMS).

To evaluate the protective efficacy of active immunization against O1, mice were pretreated by IP injection with either K2044 *magA* or K2044 *magA wbbO* (1×10^6^ CFU per dose), administered once weekly for three weeks. On the fourth week, the animals (in groups of 8) were challenged IP with a lethal dose of highly virulent *K. pneumoniae* NTUH-K2044 or NTUH-A4528. Seven of the 8 (87.5%) unimmunized control mice or mice hyperimmunized with the K2044 *magA*-mutant (K_1_^−^ O_1_) died within 19 days of NTUH-K2044 (O1:K1) infection (*P* = 0.2235, Figure 7A). Within 6 days of NTUH-A4528 (O1:K2) infection, 100% of the unimmunized control mice or the mice hyperimmunized with the K2044 *magA wbbO* double mutant (K_1_^−^ O_1_^−^) died. Meanwhile, 62.5% of the mice hyperimmunized with the K2044 *magA*-mutant (K_1_^−^ O_1_) survived without any symptoms of disease through 28 days after challenge (*P* = 0.0001, Figure 7A). Mice immunized with the K2044 *magA* mutant (K_1_^−^ O_1_) showed anti-O1 immunoglobulin G (IgG) production by immunoblot; mice hyperimmunized with the K2044 *magA wbbO* double mutant (K_1_^−^O_1_^−^) did not (Figure 7B). The results of the hyperimmunization study were consistent with a separate test in which the passive protective efficacy of O1 antiserum was tested. In the passive immunization study, mice were pretreated with naïve or immune mouse sera (negative and positive controls, respectively). The *in vivo* protective capacity of the sera was tested using a *K. pneumoniae* septicemia infection model, whereby the mice were inoculated IP with the encapsulated K2 NTUH-A4528 strain (1×10^4^ CFU). The bacterial load was determined in both the liver and spleen at 4 h post-infection. Compared to animals pre-treated with naïve serum, mice pretreated with K2044 *magA* mutant immune mouse serum (IMS-PC) had significantly reduced bacterial loads; mice pretreated with K2044 *magA wbbO* double mutant (K_1_^−^O_1_^−^) immune mouse serum (IMS-NC) showed no such reduction of bacterial load (Figure 7C). These results indicate that in both active and passive immunization studies, O1-antigen specific antiserum was able to reduce the bacterial dissemination and protect the infection of encapsulated O1:K2 PLA-associated *K. pneumoniae* in a mouse model of septicemia.

## Discussion

In contrast to the large number of capsular types (78 K types) in *Klebsiella* [24], only nine LPS O groups have been recognized, including the serotypes O1, O2, O2ac, O3, O4, O5, O7, O8, and O12 [8]. Hansen et al. used the inhibition enzyme-linked immunosorbent assay (iELISA) method to characterize 638 *K. pneumoniae* clinical isolates from Denmark, Spain, and the US. Over 90% of these screened isolates were found to be positive for O antigen, of which O1 was the most common serotype [8]. Similar serotyping of more recent isolates has been restricted by the limited availability of anti-O sera. Instead, Gierczynski et al. have tested clinical *K. pneumoniae* isolates for the presence of 7 (*waaA*, *waaE*, *waaL*, *waaQ*, *waaZ*, *waaX*, and *uge*) genes of the *waa* and 4 (*wbdA*, *wbdC*, *manB*, and *wbbO*) genes of the *wb* clusters for LPS biosynthesis [25]. No apparent correlation between genotype and serotype was observed, which is consistent with our current findings. The primers used in the present study showed a limited specificity to genes coding for biosynthesis of LPS O1. This lack of O1 specificity may reflect the genetic variation that exist in the *wb* cluster among strains of O1, O2 and O2_ac_ serotypes, since these LPS species share similar O antigenic repeats, with all containing the d-galactan I subunit [12].

The absence of the O1 antigen in the LPS of *wbbO* mutants of NTUH-K2044 or NTUH-A4528 was confirmed by silver staining and immunoblotting. In contrast to clinical *K. pneumoniae* O1 strains, the O1-antigen–deficient strain was sensitive to serum killing. This result is consistent with the previous demonstration that a high-molecular-weight fraction of *Klebsiella* O1 LPS is responsible for the resistance of the bacterium to serum killing [26]. Previous studies also reported that complement C3 binds to the O1-antigen polysaccharide units of LPS on smooth *K. pneumoniae* strains [27]. This conclusion explains why the levels of C3 complement components deposited on *wbbO* mutants were reduced compared to the wild-type parent. Removal of the O1 antigen presumably changes the sites where binding occurs, consistent with changes to the extent of opsonization [28]. In earlier studies, O1-antigen–deficient mutants were generated either in *K. pneumoniae* O1:K2 strain 52145 or 43816 by insertional mutagenesis of the *wbbM* or *wbbO* gene, respectively [5], [6]. In contrast to our results, there were no changes in serum sensitivity in those mutant strains lacking the O1 antigen [5], [6]. However, those researchers were unable to restore the wild-type phenotype of the O1-deficient mutant with a plasmid containing the *wb* cluster [5], [6], suggesting the presence of a secondary mutations in those O1-deficient mutants. The w*bbO* mutants were attenuated in *in vivo* proliferation and ability to deeply disseminate to mouse organs during the first 24 h of infection. Stimulation of proinflammatory cytokine IL-6 in mice infected with the A4528 *wbbO* mutant was decreased compared to that seen in mice infected with the parental strain. Moreover, the LD_50_ of the *wbbO* mutant was 10-fold or 200-fold higher than that of the virulent NTUH-K2044 (O1:K1) or NTUH-A4528 (O1:K2) strains, respectively, after IP injection in mice. These results suggest that the O1-deficient mutant is less virulent than the parental strain during *K. pneumoniae* infection.

Trautmann et al. found no significant differences in a comparison of the O-serotype distributions of *Klebsiella* among invasive versus noninvasive isolates [29]. Their invasive strains were obtained from blood cultures (*n* = 79), from open-lung–biopsy specimens (*n* = 7), and from patients' abdominal cavities during septic surgery (*n* = 13) [29]. In the present study, the O1 serotype represented 28.1% of clinical non–tissue-invasive *K. pneumoniae* isolates, a prevalence similar to that seen for the O1-type strains in these earlier serologic analysis of *Klebsiella* clinical isolates [8], [29]. Nonetheless, the highly prevalence (90.5%) of the O1 serotype in PLA strains indicates that the O1-type strains are predominant among *K. pneumoniae* isolates causing PLA. The prevalence of serum resistance was higher in the O1-serotype isolates than in non–O1-serotype isolates. Meanwhile, loss of the O1 antigen influenced the resistance to serum, suggesting that a correlation exists between the O1 serotype and the ability of *K. pneumoniae* to resist the bactericidal effects of human serum.

Earlier results showed that the LPS O1 antigen is masked in those *K. pneumoniae* strains harboring CPS types K1, K10, or K16; K antigen and LPS are exposed together at the cell surface in *K. pneumoniae* O1 strains harboring CPS types K2, K7, K19, K21, K22, or K66 [27]. Both of anti-O1 sera and anti-K2 sera increased the surface hydrophobicity of the O1:K2 strain, in which the K2 capsule and the O1 LPS are surface-exposed. However, anti-O1 sera did not increase the hydrophobicity of the O1:K1 strain, in which the O1 LPS was masked by the K1 capsule. Only anti-K1 sera increased the hydrophobicity of the O1:K1 strain [30]. We also found that commercial rabbit anti-K2 antisera can recognize both of the K2 and the O1 antigens. In response to infection by a virulent O1:K2 strain, high-dose pretreatment with anti-LPS O1 monoclonal antibody significantly reduced bacterial dissemination to various organs and histologic pulmonary alterations [31]. Clements et al. used *K. pneumoniae* B5055 (O1:K2), a pneumonia strain, and proved that immunization with its purified LPS can protect mice against lethal challenge with either O1:K2 or O2:K1 *K. pneumoniae* infection [32]. In our results, mice immunized with the K2044 *magA* mutant (K_1_^−^ O_1_) generated antiserum against LPS O1, and this antiserum had significantly protective efficacy against the PLA-associated O1:K2 strain, but not the PLA-associated O1:K1 strain. In both active and passive immunization studies, O1-antigen specific antiserum was able to limit the growth and reduce the bacterial dissemination of encapsulated O1:K2 PLA-associated *K. pneumoniae* in a mouse model of septicemia. Therefore, LPS O1 antigen could be a useful vaccine candidate conferring broad cross-protection against *K. pneumoniae* infection from the K2 capsular type, in which LPS O1 antigen is exposed on the bacterial surface. However, O1 antigen would not provide protection against *K. pneumoniae* infection of the capsular type (e.g., K1) that mask the O1 antigen.

In summary, LPS O1 is more prevalent in PLA strains than in non–tissue-invasive strains. The frequency of serum resistance is higher in the O1-serotype isolates than in the non–O1-serotype isolates. Mutation of the genes coding for biosynthesis of the O1 antigen changes serum resistance; reduces bacterial dissemination and colonization into deep organs, and decreases the inflammatory response to K2 PLA-associated *K. pneumoniae* infection. Immunization of mice against LPS O1 provides protection against infection with a K2 PLA-associated *K. pneumoniae* strain, but not against infection with a K1 PLA-associated *K. pneumoniae* strain.

## Materials and Methods

### Ethics statement

All animal procedures were approved under application number 20060139 of the Institutional Animal Care and Use Committee (IACUC) of the National Taiwan University College of Medicine (NTUCM). Procedures were consistent with the recommendations of the *Guide for the Care and Use of Laboratory Animals* of the National Institutes of Health and Taiwan's Animal Protection Act. These studies used BALB/cByl mice that were bred and housed in specific pathogen–free rooms within the animal care facilities of the Laboratory Animal Center at the NTUCM. Throughout the studies, mice were provided with free access to food and water.

### Bacterial strains and culture conditions

A total of 74 clinical isolates of *K. pneumoniae* were collected from 1997 to 2003 in the National Taiwan University Hospital as described previously [33]. Among the 74 isolates obtained from patients with septicemia, 42 strains were obtained from patients displaying PLA and were designated as the PLA strains. While some of these cases were complicated by metastatic meningitis and/or endophthalmitis, all incidences of PLA were confirmed by sonography-guided aspiration or surgical drainage at the time of collection. Among the remaining 32 patients who did not display clinical symptoms of liver abscess, meningitis, or endophthalmitis, diagnoses included pancreatitis, biliary tract stones with cholangitis, or gall bladder empyema; the resulting isolates were designated as non-tissue-invasive strains. Seventy-seven K-antigen *Klebsiella* reference strains were obtained from the Statens Serum Institute (Copenhagen, Denmark). *K. pneumoniae* and *E. coli* strains were cultured in Luria-Bertani (LB) medium supplemented with appropriate antibiotics, including 100 µg/mL ampicillin or 50 µg/mL kanamycin. Bacterial strains and plasmids used in this study are listed in Table S1.

### LPS-PCR genotyping

In order to detect genes coding for the LPS O1 type, polymerase chain reaction (PCR) was performed using primer pairs specific for *wbbO*, *KP3695/wbbM*, and *wzm*. Primers used in this study are listed in Table S2. PCR was performed as described elsewhere [3]. Briefly, 3 µL of overnight-cultured bacterial broth was added to 10 µL of water and boiled for 15 min to release DNA template. Reaction mixtures containing primers (0.4 µmol/L each), dNTPs (0.1 mmol/L each), *Taq* polymerase (2 U; New England Biolabs), and 13 µL of the above DNA template was incubated at 96°C for 3 min, followed by 30 cycles of 96°C for 30 s, 52°C for 15 s, and 72°C for 1 min.

### Characterization of LPS

The exopolysaccharide (EPS) extracts (containing both CPS and LPS) were purified by a modified hot water–phenol extraction method [4]. Briefly, 1 mL of bacteria cultured overnight in LB were harvested and resuspended in 150 µL of water. An equal volume of hot phenol (pH 6.6; Amresco) was added, and the mixture was vortexed vigorously. The mixture was then incubated at 65°C for 20 min, followed by chloroform extraction and centrifugation. Samples were separated by 12%-sodium dodecyl sulfate-polyacrylamide gel electrophoresis (SDS-PAGE) and then silver-stained [34]. The chemical structures of O polysaccharides in the wild-type NTUH-K2044 strain and K2044 *wbbO* mutant were analyzed by gas chromatography/mass spectrometry after derivatization with trimethylsilyl for sugar composition and with permethylated alditol acetates for sugar linkages.

### Immunoblots

The extracts of EPS from each strain were separated by 12% SDS-PAGE and blotted to a Hybond C nitrocellulose membrane (Amersham, Little Chalfont, UK). The K1 antisera were purchased from Statens Serum Institute (Denmark) and the K2 antisera were purchased from Denka Seiken (Tokyo, Japan) and diluted 1/10,000 or 1/5,000 for immunoblots. To obtain sera from K2044 *magA*-mutant hyperimmune mice (for O1 typing) or K2044 *magA wbbO* double mutant hyperimmune mice, mice were immunized intraperitoneally with 1×10^6^ colony-forming units (CFU) of K2044 *magA* mutant (K_1_^−^O_1_) or K2044 *magA wbbO* double mutant (K_1_^−^O_1_^−^). The mice were immunized three times with a single inoculation per week and were sacrificed on the fourth week. The resulting mouse sera were diluted 1/2500 or 1/500 for immunoblots.

### Serum killing assays

The survival of exponential-phase bacteria in nonimmune human serum was measured as previously described [3]. Briefly, a log-phase inoculum of 2.5×10^4^ CFU was mixed at a 1∶3 vol/vol ratio with mixed nonimmune human serum donated by 5 healthy volunteers. The final mixture, comprising 75% nonimmune serum by volume, was incubated at 37°C for 3 h. The colony count was determined by plating of serial dilutions on LB agar, and the average survival ratio was plotted. An average survival ratio ≥1 corresponds to serum resistance.

### Complement C3 deposition assays

The complement C3 deposition assay was performed by an established method [28]. Briefly, a bacterial suspension (2×10^8^ CFU/mL) was opsonized with 25% serum at 37°C for 1 min. Bacteria were washed (with centrifugation and resuspension) three times with 1× PBS. The final pellet was resuspended in 50 mM carbonate-bicarbonate buffer, pH 9.0, containing 1 M NH_4_OH, to disrupt ester bonds between C3 fragments and the bacterial surface. After 2 h at 37°C, the C3 fragment suspension was reduced with 10 mM dithiothreitol in 1% SDS at 37°C for 1 h and alkylated with 22 mM iodoacetamide at 37°C for 1 h. After 12% SDS-PAGE, the western blot analysis was performed. The membranes were incubated sequentially with anti-human complement C3 (1∶2,000; Biogenesis) and rabbit horseradish peroxidase-conjugated anti-goat IgG antibody (1∶10,000; Jackson ImmunoResearch), and developed with an enhanced chemiluminescence system (Amersham Biosciences).

### Gene deletion and complementation

*K. pneumoniae* mutated in genes coding for K antigen (*magA* or *wza wzb*), O antigen (*wbbO*), or both systems (*magA wbbO*) were constructed using the previously described unmarked deletion method [33], which employs electroporation and selection with a temperature-sensitive vector pKO3-Km containing flanking regions for each target gene. The primer pairs for the deletion constructs are listed in Table S2. For complementation, the intact *wbbO* gene was amplified by PCR and cloned into the intergenic region of the two open reading frames (ORFs), *pgpA* and *yajO*, in a pKO3-Km-*pgpA*-*yajO* recombinant vector [35]. The *wb* cluster was amplified by PCR and cloned into the PCRII-TOPO vector (Promega). The recombinant TA-*wb* plasmid was transformed into the *wbbO* mutant strains by electroporation. All of the deletion and complementation constructs were confirmed by PCR and sequence determination. All sequence data for the *K. pneumoniae* NTUH-K2044 chromosome has been deposited by Wu et al. (Genbank accession number AP006725) [36].

### Extraction and quantification of CPS

The bacterial CPS was extracted by using a method described by Domenico [37]. Briefly, 500 µl of overnight-grown bacteria was mixed with 100 µl of 1% Zwittergent 3–14 (Sigma-Aldrich, Milwaukee, WI) in 100 mM citric acid (pH 2.0) and then incubated at 50°C for 20 min. After centrifugation, 250 µl of the supernatant was transferred to a new tube, and the CPS was precipitated with 1 ml of absolute ethanol. The mixture was incubated at 4°C for 20 min. After centrifugation, the pellet was dried and dissolved in 200 µl of distilled water, and then 1,200 µl of 12.5 mM tetraborate in concentrated H_2_SO_4_ was added. The mixture was vigorously mixed and boiled for 5 min. After cooling, 20 µl of 0.15% 3-hydroxydiphenol (Sigma-Aldrich, Milwaukee, WI) was added. The tubes were shaken and the absorbance at 520 nm was measured. Uronic acid content was determined from a standard curve of d-glucuronic acid (Sigma-Aldrich, Milwaukee, WI) and expressed as micrograms per 10^9^ CFU.

### Bacterial growth assays

An 18-h culture of each strain was used to inoculate each 5-ml tube at a ratio of 1∶100. Each culture was grown at 37°C for 3 h in LB or LB supplemented with the appropriate antibiotics. After incubation, growth was monitored spectrophotometrically at 600 nm every hour.

### *Dictyostelium* phagocytosis by plaque assays

*D. discoideum* AX-2 cells were grown as previously described [22]. Briefly, *D. discoideum* AX-2 cells were grown at 23°C in HL5 medium. To test *Dictyostelium* growth on *Klebsiella* mutant strains, log phase cultured bacteria were plated on SM agar. Doses of 1200, 2500, or 5000 *Dictyostelium* cells were added to the bacterial lawn, and the formation of phagocytic plaques was observed after 5 days.

### Mouse inoculation experiments

#### Virulence assay

Virulence was evaluated by mortality in two different models: a murine model of septicemia generated by IP injection, and a murine model of liver abscess generated by intragastric injection. Groups of five-week-old female BALB/cByl mice were infected IP or intragastrically with isogenic *K. pneumoniae* NTIH-K2044 or NTUH-A4528 mutants in 0.1 mL of 0.95% saline (10^2^–10^7^ CFU; 4 mice for each dose). The exact inoculation dose was confirmed by serial dilution and plating to LB agar. Mice were monitored for 4 or 6 weeks in IP or intragastric inoculation experiments (respectively); upon death or after sacrifice of surviving mice (at the end of the 4 or 6 weeks), the liver and brain were removed for histopathologic examination. The 50% lethal dose (LD_50_) was calculated as described by Reed and Muench [38]. To determine the bacterial load *in vivo*, the same inoculation dose (1×10^3^ CFU) of each strain (4 mice for each group) was administered by IP injection. Surviving animals were sacrificed at 24 hours after challenge; blood and organ homogenates (including liver and spleen) were cultured for quantification of CFU. The number of CFU detected in the organs was standardized per 0.1 gram wet organ weight. Sera were collected at 24 h; IL-6 and TNF-alpha levels were measured by ELISA (R&D Systems, Minneapolis, MN).

#### Protection study

For active immunization, five-week-old BALB/cByl mice were immunized three times by once-weekly IP injection with the indicated dose of the lived K2044 *magA*-mutant or K2044 *magA wbbO* double mutant, and were challenged at the fourth week. Age-matched, unimmunized control mice were inoculated with saline (8 mice per group). After 4 weeks, immunized and unimmunized control mice were challenged with 1×10^3^ CFU of NTUH-K2044 (O1:K1) or NTUH-A4528 (O1:K2); this dose is greater than the LD_50_ value. The challenged mice were observed for 28 days for mortality and clinical signs. Survival was analyzed by Kaplan-Meier analysis with a log-rank test; a *P* value<0.05 was considered to be statistically significant. For passive protection, 100 µL of immunized and unimmunized control mice sera was administered to five-week-old BALB/cByl mice by IP injection. One hour after serum injection, the mice were infected by intraperitoneal inoculation with 1×10^4^ CFU of NTUH-A4528 (O1:K2). Four hour after infection, the mice were euthanized and bacterial counts from liver and spleen were determined.

### Statistical Analyses

Data are presented as means ± standard deviations (SDs). Statistical significance of comparisons of mean values was assessed by a two-tailed Student's *t* test using Prism 5 (Graphpad) software. Prevalence was analyzed by chi-square test using SPSS version 12.0 software. Survival was analyzed by Kaplan-Meier analysis with a log-rank test. *P* values of <0.05 were considered significant.

## Supporting Information

Table S1 Bacterial strains and plasmids used in this study
(DOCX)

Table S2 Primers used in this study
(DOCX)
