# Supplementary material for: Lipopolysaccharide O1 Antigen Contributes to the Virulence in Klebsiella pneumoniae Causing Pyogenic Liver Abscess
Source: PLoS One. 2012 Mar 12;7(3):e33155. doi: 10.1371/journal.pone.0033155 (PMC3299736; doi:10.1371/journal.pone.0033155)
Supplement: Table S1 — Bacterial strains and plasmids used in this study (DOCX) [file pone.0033155.s001.docx]

**Table S1. Bacterial strains and plasmids used in this study**

| Bacterial strain or plasmid | Genotype and relevant description* | Reference or  source |
| --- | --- | --- |
| **Bacteria** |  |  |
| *Klebsiella pneumoniae* strains | | |
| PLAstrain (42 strains) | Clinical isolate *K. pneumoniae* strains causing PLA and collected from NTUH during 1997-2003. | [[33](#_ENREF_33),[35](#_ENREF_35)] |
| Non–tissue-invasive strain (32 strains) | Clinical isolate *K. pneumoniae* strains causing pancreatitis, biliary tract stones with cholangitis or gall bladder empyema and collected from NTUH during 1997-2003. | [[33](#_ENREF_33),[35](#_ENREF_35)] |
| NTUH-K2044 | O1:K1, Sr, clinical isolate PLA strain, the parent K1 strain for generate isogenic mutants | [[3](#_ENREF_3)] |
| K2044 ΔwbbO | NTUH-K2044 isogenic mutant with deletion of *wbbO* gene | This study |
| K2044 ΔwbbO::wbbO | K2044 ΔwbbO with *wbbO* cassette between *pgpA* and *yajO* | This study |
| K2044 ΔwbbO::TA-wb | K2044 ΔwbbO cotaining TA-*wb* plasmid | This study |
| K2044 ΔmagA | NTUH-K2044 isogenic mutant with deletion of *magA* gene | This study |
| K2044 ΔmagA wbbO | NTUH-K2044 isogenic mutant with double deletion of *magA* and *wbbO* genes | This study |
| NTUH-A4528 | O1:K2, Sr, clinical isolate PLA strain, the parent K2 strain for generate isogenic mutants | [[3](#_ENREF_3)] |
| A4528 ΔwbbO | NTUH-A4528 isogenic mutant with deletion of *wbbO* gene | This study |
| A4528 ΔwbbO::TA-wb | A4528 ΔwbbO cotaining TA-*wb* plasmid | This study |
| A4528 Δwza wzb | NTUH-A4528 isogenic mutant with deletion of *wza* and *wzb* genes | This study |
| *Escherichia coli* strains | | |
| DH10B | F– *mcrA* Δ*(mrr-hsdRMS-mcrBC)* *80* Z ΔM15Δ*lacX74 endA1 recA1 deoR (ara leu)7697 araΔ139 galU galK nupG rpsL* – | Invitrogen |
| **Plasmids** |  |  |
| pGEM-T Easy | TA cloning vector | Promega |
| PCRⅡ-TOPO | TA cloning vector | Invitrogen |
| pKO3-Km | pKO3 derived plasmid, with an insertion of Km resistance cassette from pUC4K into AccI site | [[33](#_ENREF_33)] |
| pKO3-Km-*pgpA*-*yajO* | pKO3 derived plasmid, with the *pgpA*-*yajO* DNA fragments for cis-complementation | [[35](#_ENREF_35)] |
| TA-*wb* | PCRⅡ-TOPO TA cloning vector carrying with the *wb* cluster from NTUH-K2044 | This study |
| TA-GFP | PCRⅡ-TOPO TA cloning vector carrying with GFP | [[3](#_ENREF_3)] |

NOTE. PLA, pyogenic liver abscess; NTUH, National Taiwan University Hospital; Km, kanamycin; Sr, resistance; Ss, sensitive; GFP, green fluorescence protein
