# Supplementary material for: Lipopolysaccharide O1 Antigen Contributes to the Virulence in Klebsiella pneumoniae Causing Pyogenic Liver Abscess
Source: PLoS One. 2012 Mar 12;7(3):e33155. doi: 10.1371/journal.pone.0033155 (PMC3299736; doi:10.1371/journal.pone.0033155)
Supplement: Table S2 — Primers used in this study (DOCX) [file pone.0033155.s002.docx]

**Table S2. Primers used in this study**

| Primers | Sequence (5’ → 3’) | Purpose | Source |
| --- | --- | --- | --- |
| wbbO-F | gtgggcatagcaatgagaaaattg | O1-serotype *wbbO* PCR genotyping | This study |
| wbbO-R | cgtgctttaggattgctaagc | O1-serotype *wbbO* PCR genotyping | This study |
| wzm-F | Cagctgacctgataaactttgc | O1-serotype *wzm* PCR genotyping | This study |
| wzm-R | ttacaagatctctgcaaatc | O1-serotype *wzm* PCR genotyping | This study |
| KP3695(-1)R | ctgttgtctcttttattatcc | O1-serotype *wbbM* PCR genotyping | This study |
| KP3696(+902)F | attccattgtccggcatgcg | O1-serotype *wbbM* PCR genotyping | This study |
| 5-mucA-F | GCAATCTAATCTGCCACCTG | K2044 *magA* deletion mutant construct | This study |
| 3-mucA-R | CACCAGTGGATTTAGAGGAC | K2044 *magA* deletion mutant construct | This study |
| 601R | CACTTCTCGTATTTGCGGCG | K2044 *magA* deletion mutant construct | This study |
| magA-F | gtaatttagaaaagggacag | K2044 *magA* deletion mutant construct | This study |
| KP3695(+1152)F | atgaaatatacggcattgatag | *wbbO* deletion mutant construct | This study |
| wbbO-RR | cagcgattcgtcgataagcagaaag | *wbbO* deletion mutant construct | This study |
| wbbO-IF | tgtgtaagtttcacatttattattgcg | *wbbO* deletion mutant construct | This study |
| wbbO-IR | ttaatgatacttaccactaataccttttatgcc | *wbbO* deletion mutant construct | This study |
| wzm-p | catgctgtccttagatggttc | *wbbO* complementation construct | This study |
| wbbO-R1 | tcatcgaactacatcatgatatatttgc | *wbbO* complementation construct | This study |
| wzm-IR | aaattattctgcaaatatccactgaatgag | *wbbO* complementation construct | This study |
| wbbO-rbs | ggcataaaaggtattagtggtaag | *wbbO* complementation construct | This study |
| wzm-p | catgctgtccttagatggttc | *wb* cluster complementation construct | This study |
| wbbO-R1 | tcatcgaactacatcatgatatatttgc | *wb* cluster complementation construct | This study |
| wza-CF | gtgaagttctggaaccagtgg | A4528 *wza wzb* deletion mutant construct | This study |
| K2 wzc-R6 | tataatcaaagccctctgtctg | A4528 *wza wzb* deletion mutant construct | This study |
| K2 wzab-IR | aatgtcacatcatcagtaaatc | A4528 *wza wzb* deletion mutant construct | This study |
| K2 wzab-IF | aaattcaggaaataatgcatgac | A4528 *wza wzb* deletion mutant construct | This study |
